# Mortality in patients with normal left ventricular function requiring emergency VA-ECMO for postcardiotomy cardiogenic shock due to coronary malperfusion

**DOI:** 10.1371/journal.pone.0300568

**Published:** 2024-03-21

**Authors:** Jagdip Kang, Mateo Marin-Cuartas, Anna Flo Forner, Priya R. Menon, André Ginther, Diyar Saaed, Suzanne de Waha, Massimiliano Meineri, Jörg Ender, Michael A. Borger

**Affiliations:** 1 Department of Cardiac Surgery, Leipzig Heart Center, Leipzig, Germany; 2 Department of Anesthesiology and Intensive Care, Leipzig Heart Center, Leipzig, Germany; Tehran University of Medical Sciences, ISLAMIC REPUBLIC OF IRAN

## Abstract

**Objectives:**

To analyze outcomes in patients with normal preoperative left ventricular ejection fraction (LVEF) undergoing venoarterial extracorporeal membrane oxygenation (VA-ECMO) therapy due to postcardiotomy cardiogenic shock (PCCS) related to coronary malperfusion.

**Methods:**

Retrospective single-center analysis in patients with normal preoperative LVEF treated with VA-ECMO for coronary malperfusion-related PCCS between May 1998 and May 2018. The primary outcome was 30-day mortality, which was compared using the Kaplan-Meier method and the log-rank test. Multivariable logistic regression was performed to identify predictors of mortality.

**Results:**

During the study period, a total of 62,125 patients underwent cardiac surgery at our institution. Amongst them, 59 patients (0.1%) with normal preoperative LVEF required VA-ECMO support due to coronary malperfusion-related PCCS. The mean duration of VA-ECMO support was 6 days (interquartile range 4–7 days). The 30-day mortality was 50.8%. Under VA-ECMO therapy, a complication composite outcome of bleeding, re-exploration for bleeding, acute renal failure, acute liver failure, and sepsis occurred in 51 (86.4%) patients. Independent predictors of 30-day mortality were lactate levels > 9.9 mmol/l before VA-ECMO implantation (odds ratio [OR]: 3.3; 95% confidence interval [CI] 1.5–7.0; p = 0.002), delay until revascularization > 278 minutes (OR: 2.9; 95% CI 1.3–6.4; p = 0.008) and peripheral arterial artery disease (OR: 3.3; 95% 1.6–7.5; p = 0.001).

**Conclusions:**

Mortality rates are high in patients with normal preoperative LVEF who develop PCCS due to coronary malperfusion. The early implantation of VA-ECMO before the development of profound tissue hypoxia and early coronary revascularization increases the likelihood of survival. Lactate levels are useful to define optimal timing for the VA-ECMO initiation.

## Introduction

Postcardiotomy cardiogenic shock (PCCS) is a devastating complication after cardiac surgery occurring in 0.5% - 2% of all patients undergoing cardiac surgery with reported mortality rates of up to 76% [1, 2]. Patients developing PCCS have limited medical treatment options besides optimal fluid management, vasopressors and inotropic agents. Therefore, temporary mechanical circulatory support (MCS) with venoarterial extracorporeal membrane oxygenation (VA-ECMO) is crucial in managing these patients. Despite the well-established use of VA-ECMO in cardiac surgery, the rates of weaning failure as well as early and late mortality in patients with PCCS are still high [3]. The decision for initiation of VA-ECMO is difficult, considering the unexpected occurrence of PCCS in patients with preoperative normal left ventricular ejection fraction (LVEF) and the poor outcomes despite the timely initiation of MCS. Clinical management and patient selection are even more challenging in the particular subset of PCCS related to postoperative coronary malperfusion due to the paucity of data supporting decision-making in this specific clinical setting.

Hence, this study aims to analyze the early outcomes of patients with normal preoperative LVEF with perioperative coronary malperfusion requiring temporary MCS with VA-ECMO due to PCCS within the first 48 postoperative hours. Furthermore, this study aims to identify predictors of early mortality in this patient cohort to help surgeons and intensivists in their decision-making process. We hypothesize that early initiation of VA-ECMO and prompt coronary revascularization improve the outcomes of patients with PCCS due to coronary malperfusion.

## Methods

### Study design

This study was approved by the ethics committee at the University of Leipzig (protocol number 391/18-ek), and individual patient informed consent was waived. During data collection, the authors had access to patient-specific information which enabled the identification of every patient to collect the necessary data and perform follow-up. After data collection, the patient cohort was analyzed anonymously. Between May 1998 and May 2018, all adult patients with normal preoperative LVEF requiring temporary MCS with VA-ECMO due to coronary malperfusion within 48 hours after cardiac surgery at our center were included in the analysis. LVEF was determined through preoperative echocardiography. Patients under 18 years, male patients with LVEF of less than 52%, and female patients with LVEF of less than 54% were excluded from this study [4]. The primary outcome was 30-day postoperative mortality. The secondary outcome was a complication composite outcome of bleeding, re-exploration for bleeding, acute renal failure, acute liver failure, and sepsis.

### Data collection and definitions

The demographic profile of patients, intraoperative data, postoperative outcomes, and specific information related to the VA-ECMO (indication, cannulation site, etc.) were prospectively collected and entered our computerized institutional database from May 1998 to May 2018. Data collection and follow-up was performed until January 2022. The data were retrospectively analyzed.

Delay until revascularization was defined as the time elapsed between weaning off from cardiopulmonary bypass (CPB) and revascularization. Delay until VA-ECMO implantation was defined as the time elapsed between weaning off from CPB and VA-ECMO implantation. Coronary malperfusion was detected either by intraoperative bypass flow measurement using transit-time flow measurement or postoperative coronary angiography. The definitions of additional study variables are summarized in the **S1 Data**.

### VA-ECMO circuit and implantation techniques

In most cases, the VA-ECMO system components consisted of a SCPC-centrifugal pump console (LivaNova PLC, London, UK), a Revolution centrifugal pump (LivaNova PLC), one A.L.ONE ECMO oxygenator (EUROSETS, Milano, Italy), and an HLS cannula set (containing arterial cannulas ranging from 17–19 Fr and venous cannulas ranging from 19–23 Fr (Maquet GmbH—Getinge AB, Rastatt, Germany). The tubing set was covered with heparin in a standard fashion.

Implantation of peripheral VA-ECMO was performed either using a percutaneous Seldinger´s technique with ultrasound-guided puncture of the femoral artery and vein or under direct surgical preparation of the femoral vessels depending on the clinical status and previous medical history of the patient. If the decision was made for central VA-ECMO, venous cannulation was performed via the femoral vein, and arterial cannulation was performed via an 8 mm Dacron prosthesis anastomosed either to the right axillary artery or to the ascending aorta. Depending on the patient’s clinical status, the sternum was either primarily closed after cannulation of the ascending aorta or left open until the patient stabilized hemodynamically. The size of the VA-ECMO cannulas was selected according to the patient’s body surface area. Transesophageal echocardiography was always performed during the procedure to confirm the accurate placement of the cannulas.

### Statistical analysis

Categorical variables are expressed as frequencies and percentages throughout the manuscript. Continuous variables are expressed as mean ± standard deviation for normally distributed variables and median and interquartile range (IQR) for non-normally distributed variables. For normally distributed variables, the student’s *t*-test was used. The Mann–Whitney *U*-test was used for non-normally distributed variables. The chi-squared or Fisher’s exact test, as appropriate, was performed for categorical variables. The Kaplan-Meier method was used to obtain non-parametric estimates of all-cause 30-day mortality and compared with the log-rank test. Univariate analysis was performed to determine clinical predictors of early mortality. Perioperative variables with a univariable value of p < 0.25 or those judged clinically important were included in a multivariable logistic regression model. We included following preoperative variables: peripheral vascular disease, hypertension, gender, redo surgery, logistic EuroScore, creatine clearance and following variables prior VA-ECMO implantation: creatine clearance, creatine kinase isoenzyme MB concentration, lactate level, hematocrit, aspartate transaminase concentration, usage of terlipressin, mean arterial pressure and time to revascularization. The multivariable logistic regression model with the variable ‘survival’ as the dependent variable was constructed using a backward stepwise selection procedure. Potential predictors were removed if this exclusion did not result in a significant change in the log-likelihood ratio test. The cut-off for variable removal was set at a significance level of 0.05. To avoid overfitting and obtain reliable internal validation of the subset of factors, a bootstrap method was used, which derived 1000 computer-generated samples by random selection with replacement, each including the same number of patients. Within each bootstrap sample, the B-coefficient was calculated using all selected independent variables. The robustness of the model and thus the reliability of predictor variables in the final regression model were estimated by the 95% confidence interval [CI] of the B-coefficient derived from the bootstrap samples. To consider the continuous variables “lactate level before VA-ECMO” and “time to revascularization” as prognostic markers, a cut-off-point was determined by performing a receiver operating characteristics (ROC) analysis to determine the optimal sensitivity and specificity of the variable values. The point closest to the corner in the ROC plane was defined as the optimal cut-off point. Statistical analyses were performed using the SPSS software package, version 25.0 (IBM Corp, Armonk, NY, USA). Bootstrapping was performed using R, version 3.4.4 (R Foundation for Statistical Computing, Vienna, Austria).

## Results

### Patients

In a total study period of 20 years (May 1998 –May 2018), a total of 62,125 adult patients underwent cardiac surgery at our institution. Among them, 59 patients (0.1%) with normal preoperative LVEF required VA-ECMO within the first 48 hours after presenting with PCCS due to coronary malperfusion. These patients form the basis of the present study.

### Demographic characteristics and intraoperative data

Preoperative patient characteristics are shown in **Table 1**. The type of surgery and intraoperative details are depicted in **Table 2**. Clinical and laboratory parameters immediately before VA-ECMO initiation are summarized in **Table 3**.

**Table 1:** Baseline patient characteristics.

Variable	Survivors (n = 29)	Non-survivors (n = 30)	p-value
Age–years	63.1 (58.1–68.1)	65.6 (61.6–69.7)	0.43
Male gender	18 (62.1)	12 (40.0)	0.09
LVEF—%	62 (±5)	60 (±5)	0.40
Arterial hypertension	19 (65.5)	26 (86.7)	0.07
Pulmonary hypertension	6(23.1)	7 (23.3)	0.98
COPD	1 (3.4)	3 (10.0)	0.61
Smoker	10 (38.5)	9 (31.0)	0.56
Hyperlipidemia	13 (46.4)	14 (46.7)	0.99
BMI—kg/m^2^	26.6 (24.8–28.4)	26.6 (24.9–28.3)	0.69
Diabetes mellitus	9 (31.0)	12 (40.0)	0.47
Peripheral artery disease	6 (20.7)	15 (50.0)	0.02
Neurological dysfunction	5 (17.2)	5 (16.7)	0.95
Creatinine—mg\dl	0.9 (0.8–1.1)	1.3 (1.1–1.4)	0.02
GFR—ml\min	94.3 (73.5-115-1)	62.9 (53.7–72.3)	0.006
Preoperative dialysis	0 (0)	2 (6.7)	0.49
Elective surgery	19 (65.5)	20 (66.7)	0.93
Urgent surgery	5 (17.2)	6 (20.0)	0.79
Emergent surgery	5 (17.2)	4 (13.3)	0.68
Left main stenosis	2 (11.8)	3 (25.0)	0.35
Prior PCI	5 (17.2)	7 (23.3)	0.56
Prior MI	6 (20.7)	7 (23.3)	0.92
Prior heart surgery	5 (17.2)	10 (33.3)	0.16
Logistic EuroSCORE—%	6.2 (2.8–11.4)	10.2 (6.0–20.6)	0.02
Active endocarditis	2 (6.9)	2 (6.7)	1.00
Type A aortic dissection	0 (0)	1 (3.3)	1.00

Categorical variables expressed as number (percentage), continuous variables expressed as median (interquartile range) or mean (standard deviation); **BMI** Body mass index; **COPD** Chronic obstructive pulmonary disease; **GFR** Glomerular filtration rate; **LVEF** Left ventricular ejection fraction; **MI** Myocardial infarction; **PCI** Percutaneous coronary intervention.

**Table 2:** Intraoperative data.

Variable	Survivors (n = 29)	Non-survivors (n = 30)	p-value
*Type of surgery*			
On pump CABG	7 (24.1)	3 (10.0)	0.18
OPCAB	4 (13.8)	2 (6.70)	0.42
CABG + valve surgery	5 (17.2)	3 (10.0)	0.47
Isolated AVR	0 (0)	4 (13.3)	0.11
AVR + AAR	3 (10.3)	3 (10.0)	1.00
Bentall	5 (17.2)	6 (20.0)	1.00
Aortic arch surgery	1 (3.4)	2 (6.7)	1.00
MVR + TVR	3 (10.3)	6 (20.0)	0.47
AVR + MVR/TVR	1 (3.4)	1 (3.3)	1.00
*Intraoperative variables*			
Length of surgery—min	257 (152–387)	245 (165–362)	0.75
Bypass time—min	127 (98–217)	134 (92–203)	0.82
Cross clamp time—min	97 (71–115)	89 (62–111)	0.32
Minimal temperature—Celsius	34 (32–35)	34 (31–34)	0.50

Categorical variables expressed as number (percentage), continuous variables expressed as median (interquartile range); **AAR** Ascending aortic replacement; **AVR** Aortic valve replacement; **CABG** Coronary artery bypass grafting; **OPCAB** Off-pump coronary artery bypass; **MVR** Mitral valve replacement; **TVR** Tricuspid valve replacement

**Table 3:** Perioperative parameters immediately before VA-ECMO implantation.

Variable	Survivors (n = 29)	Non-survivors (n = 30)	p-value
Lactate—mmol\l	6.5 (5.1–7.9)	11.2 (8.9–13.4)	0.001
pH	6.8 (6.1–7.6)	7.1 (6.6–7.6)	0.50
Glucose—mmol\l	10.1 (8.7–11.6)	10.1 (8.7–11.4)	0.93
Haematocrit—%	30.3(28.6–31.9)	27.2 (25.6–28.8)	0.009
Systolic blood pressure—mmHg	79.9 (74.1–85.8)	75.7 (71.8–79.7)	0.20
Diastolic blood pressure—mmHg	47.0 (43.9–50.1)	39.3 (36.9–41.7)	<0.001
Mean blood pressure—mmHg	57.9 (54.8–61.2)	51.4 (49.1–53.7)	0.001
CK before VA-ECMO - μmol/L	9.9 (3.7–31.6)	19.9 (11.1–35.9)	0.21
CKMB before VA-ECMO - μmol/L	1.2 (0.6–5.1)	2.8 (1.9–6.1)	0.08
ASAT- μmol/L	1.6 (0.8–5.7)	4.5 (2.3–11.1)	0.30
ALAT- μmol/L	1.2 (0.3–2.1)	1.6 (0.9–2.3)	0.60
INR	1.5 (1.3–1.7)	1.7 (1.3–1.9)	0.49
Creatinine—mg\dl	1.7 (1.3–2.1)	2.5 (2.1–2.8)	0.005
GFR—ml\min	58.7 (45.2–72.3)	30.2 (26.1–34.5)	<0.001
Epinephrine—ml\h	30 (14–50)	31.5 (10–53.7)	0.96
Norepinephrine—ml\h	35 (18–50)	35 (20–80)	0.54
Telipressin (1mg/10ml)—ml\h	0 (0–0)	0 (0–11)	0.04
Dobutamine (250mg/50ml)—ml\h	0 (0–3)	0 (0–1.5)	0.84
Milrinone (10mg/50ml)—ml\h	0 (0–3.0)	0 (0–2.9)	0.71
IABP—n (%)	13 (44.8)	19 (63.3)	0.20

Categorical variables expressed as number (percentage), continuous variables expressed as median (interquartile range); **ALAT** Alanine transaminase; **ASAT** Aspartate transaminase; **CK** Creatine kinase; **CKMB** Creatine kinase-MB; **GFR** Glomerular filtration rate; **IABP** Intra-aortic balloon pump; **INR** International normalized ratio

### VA-ECMO therapy

The delay until revascularization was significantly longer in the non-survivor group with 470 minutes (IQR 112–1489 minutes) vs. 180 minutes (IQR 0–608 minutes) in the survivors’ group (p = 0.04). VA-ECMO-related complications occurred in 86.4% of patients. Renal failure occurred in 28 (47.5%) patients and bleeding in 44 (74.6%) patients (**Table 4**). VA-ECMO-related complications occurred with a similar incidence in both groups. The type of surgery did not influence survival.

**Table 4:** VA-ECMO specific details and early postoperative complications.

Variable	Survivors (n = 29)	Non-survivors (n = 30)	p-value
Antegrade cannulation	16 (55.2)	19 (63.3)	0.52
Retrograde cannulation	13 (44.8)	11 (36.7)	0.52
Delay until revascularization* - minutes	180 (0–608)	470 (112–1489)	0.047
Delay until VA-ECMO implantation** - minutes	180 (7–558)	425 (0–2105)	0.14
Time on VA-ECMO—days	6 (5–7)	6 (4–7)	0.87
Complication composite outcome***	23 (79.3)	28 (93.3)	0.15
Bleeding	19 (65.5)	25 (83.3)	0.12
Re-exploration for bleeding	12 (41.4)	9 (30.0)	0.36
Acute renal failure	13 (44.8)	15 (50.0)	0.69
Acute liver failure	0 (0)	3 (10)	0.24
Sepsis	2 (6.9)	2 (6.7)	1.00
Stroke	1 (3.4)	6 (20)	0.10
Thrombosis	1 (3.4)	2 (6.7)	1.00

Categorical variables expressed as number (percentage), continuous. Variables as median (interquartile range); **VA-ECMO** Venoarterial extracorporeal membrane oxygenation

* defined as the time between cardiopulmonary bypass (CPB) weaning and revascularization

** defined as the time between CPB weaning and VA-ECMO implantation

*** Complication composite outcome of bleeding, re-exploration for bleeding, acute renal failure, acute liver failure and sepsis

### Postoperative outcomes

From a total of 59 patients, 47 patients (79.7%) could be weaned off from VA-ECMO. The median time on VA-ECMO was 6 days (IQR 4–7 days). None of the patients received escalated mechanical circulatory support therapy in the form of a left ventricular assist device (LVAD) or Impella. A total of 12 (20.3%) patients died on VA-ECMO, with a median survival of 6 days (IQR 4–7 days). Amongst the 47 patients who could be weaned off from VA-ECMO, 29 (61.7%) patients were alive at 30 days. The 30-day survivors had a median hospital stay of 24 days (IQR 12–98 days), and non-survivors had a median hospital stay of 8 days (IQR 1–112 days). The overall 30-day mortality was 50.8%. Those patients who survived the 30-day mark had a median survival of 408 days (IQR 87 days– 6 years). Among the 27 patients who survived the 30-day mark, at one year, 6 (22.2%) patients were lost to follow-up, 7 (25.9%) patients died, and 14 (51.9%) were alive.

**Fig 1:**
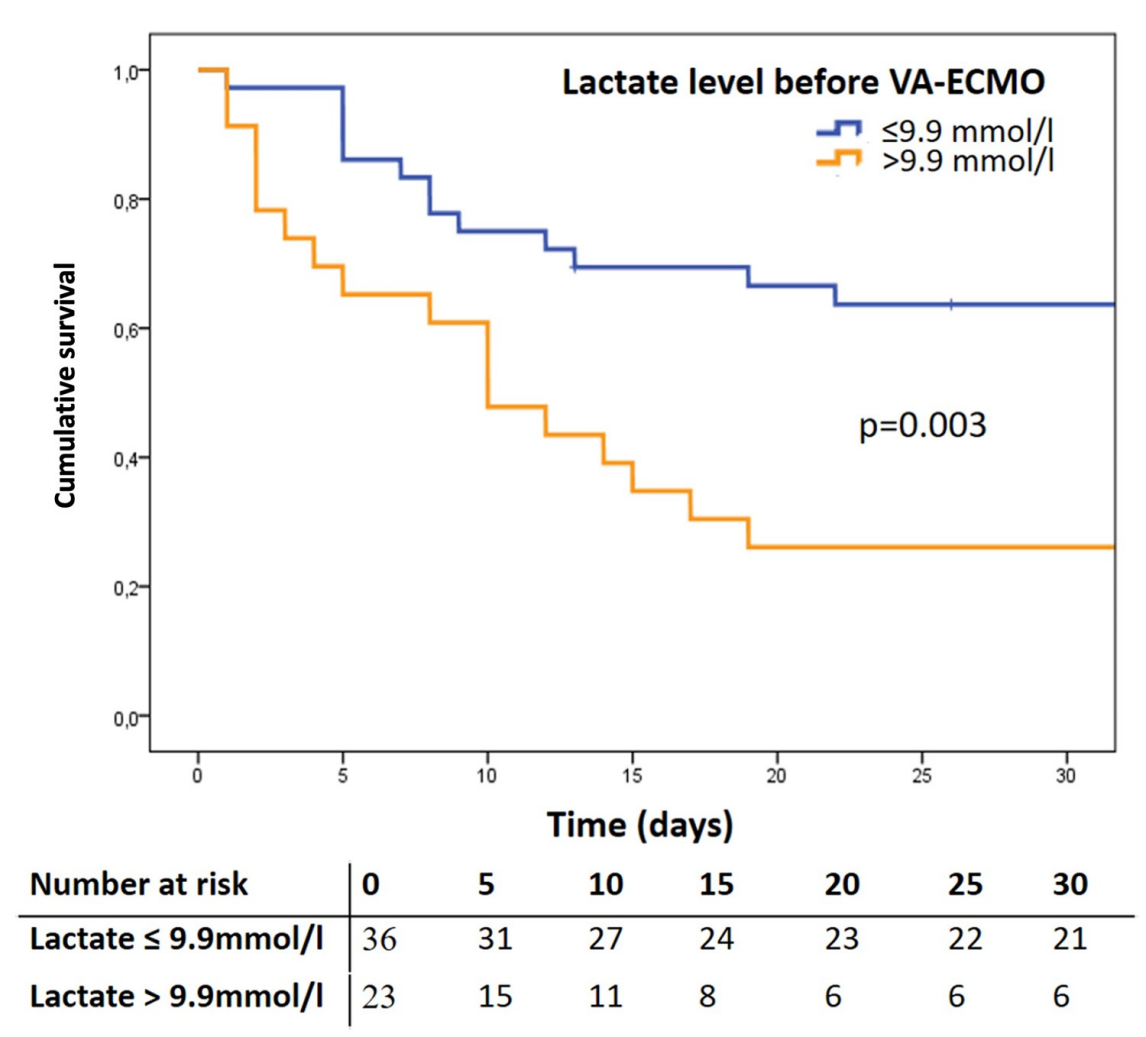
Kaplan Meier survival curves according to lactate level cut-off before VA-ECMO implantation.

**Fig 2:**
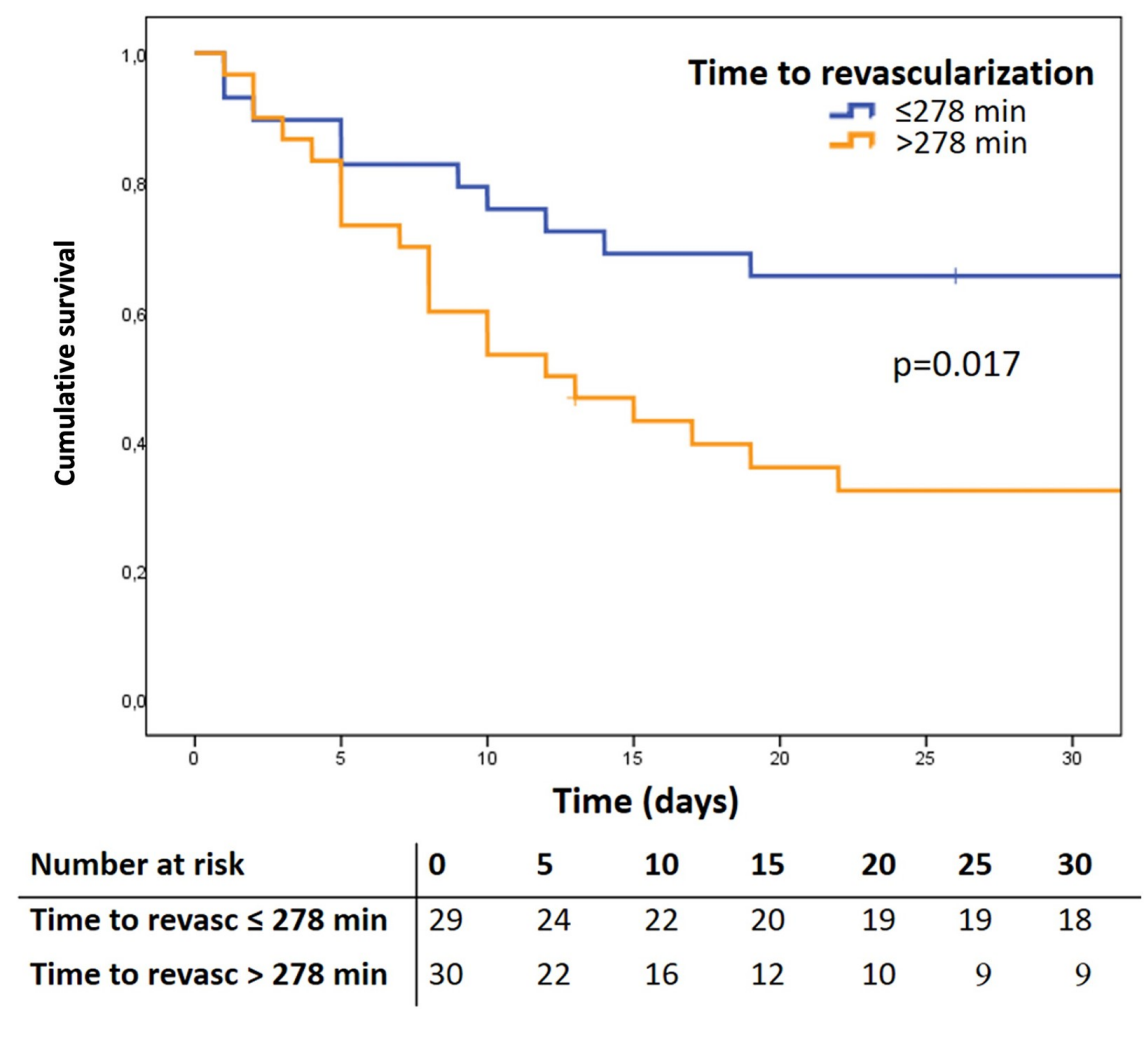
Kaplan Meier survival curve according to the delay time cut-off until revascularization.

Independent predictors of 30-day mortality were peripheral artery disease, high lactate level before VA-ECMO implantation (cut-off > 9.9 mmol/l) (**Fig 1**), and delay until revascularization (cut-off > 278 minutes) (**Fig 2**). The results of the multivariable regression analysis are summarized in **Table 5**.

**Table 5:** Independent predictors of 30-day mortality.

Preoperative parameters	Odds ratio	95% CI	p-value
Peripheral artery disease	3.5	1.6–7.5	0.001
Arterial lactate before VA-ECMO > 9.9 mmol/l	3.3	1.5–7.0	0.002
Time until revascularization > 278 min	2.9	1.3–6.4	0.008

**CI** Confidence interval, **VA-ECMO** Veno-arterial extracorporeal membrane oxygenation, **CPB** Cardiopulmonary bypass

## Discussion

The current study analyzes the 30-day mortality in patients with preoperative normal LVEF who developed PCCS due to coronary malperfusion treated by MCS with VA-ECMO. The 30-day mortality in this patient cohort was 50.8%, and a complication composite outcome of bleeding, re-exploration for bleeding, acute renal failure, acute liver failure, and sepsis occurred in 86.4% of the patients. In addition, we identified high lactate levels (cut-off > 9.9 mmol/l) before VA-ECMO implantation, delayed revascularization (> 278 minutes), and peripheral vascular disease to be independent predictors of 30-day mortality.

Refractory PCCS is a highly lethal complication in cardiac surgery with a high likelihood of death without further intervention. The high 30-day mortality in our cohort again highlights the devastating nature of PCCS. Mortality rates are still high even under maximal therapeutic interventions including VA-ECMO [5]. Coronary malperfusion is the most common cause of PCCS [5]. Our institution’s management strategy for patients with PCCS due to coronary malperfusion is early revascularization and temporary MCS with VA-ECMO. If a patient presents with clinical signs of coronary malperfusion postoperatively, our standard is to perform early coronary angiography based on the widely adopted principle of “time is myocardium”. In the case of angiographically confirmed coronary malperfusion, the decision on how to revascularize the patient is based on a multidisciplinary heart team decision. Aspects such as type of surgery, severity of hemodynamic instability, active bleeding, age, and life expectancy are considered within the decision process. Once a treatment strategy is defined, revascularization is performed as soon as possible, aiming to allow rapid recovery of as much stunned myocardium as possible. Further, the need for temporary MCS is assessed. However, due to the multifactorial and complex nature of refractory PCCS, the ideal timing to initiate MCS is challenging. Hence, the identification of preoperative indicators for mortality is essential to help surgeons and intensivists in this complex clinical scenario. The fact that we identified high lactate levels at the time of VA-ECMO initiation as an independent predictor of mortality indicates that early diagnosis and timely management of PCCS before profound tissue hypoxia occurs are of great importance to limit end-organ damage and increase the probability of survival. Our results confirmed our hypothesis, indicating that early initiation of VA-ECMO and prompt coronary revascularization improve the outcomes of patients with PCCS due to coronary malperfusion.

In addition to these predictors, a history of peripheral artery disease was identified as a non-modifiable independent predictor of early mortality. Peripheral artery disease is a surrogate of high atherosclerotic burden, which most likely explains its predictive value for poor outcomes. In addition, although non-modifiable, peripheral artery disease is relevant during the planning of the VA-ECMO implantation since using alternative cannulation strategies could prevent possible complications such as limb ischemia. In the case of peripheral cannulation for VA-ECMO, we routinely implant an ipsilateral antegrade perfusion cannula to grant leg perfusion. Moreover, close monitoring of limb perfusion should be performed in all patients during the post-implantation period in the ICU. Additional risk factors such as age, gender, and type of surgery, which were identified as predictors of mortality in other studies [6–8], did not influence mortality in our patient cohort. Hence, we consider that age alone is insufficient to contraindicate the initiation of VA-ECMO therapy. We consider MCS with VA-ECMO in elderly patients to be reasonable if the patient presents with unexpected PCCS due to coronary malperfusion and does not have severe comorbidities that negatively influence their life expectancy.

In our series, the median duration of VA-ECMO therapy was 6 days and was similar among those patients who survived and those patients who did not survive. This finding is in line with other studies and indicates that patients developing PCCS should receive VA-ECMO support for at least 4 to 7 days [9]. However, more extended VA-ECMO support does not provide any survival advantage. Therefore, we consider that the time on VA-ECMO should be limited in patients in whom weaning off seems to be unlikely [5]. An escalation of mechanical circulatory support should be considered in the patient subset where weaning of VA-ECMO is futile and no contraindications for definitive mechanical circulatory support exist. In a small study performed by *Bertoldi et al*., 9 patients received Impella 5.0 support after initial VA-ECMO and Impella 2.5/CP support. After a median Impella 5.0 support of 17 days, all patients underwent LVAD implantation with a survival rate of 88.9% at a median follow-up time of 227 days [10].

As reported by *Raffa et al*. [11], we did not observe any difference in complication rates according to the site of VA-ECMO cannulation (i.e., peripheral or central). *Hess et al*. [12] reported postoperative mortality rates of 29.8% in a comparable patient population requiring postcardiotomy MCS support due to different PCCS etiologies. In our patients´ cohort with coronary malperfusion as a cause of PCCS, the 30-day mortality was considerably higher (50.8%). This indicates that coronary malperfusion that leads to PCCS still has a high lethality, despite the advances in its treatment that occurred during the study period. Consequently, we believe that rather than further technological advances, the most effective—and simple- way to improve outcomes in these critically ill patients is to timely provide MCS with VA-ECMO and early suspect, detect and treat coronary malperfusion.

### Study limitations

The main limitation of this study lies in its retrospective and single-center nature and the related biases thereto. The implantation technique and cannulation site were defined at the surgeon’s discretion. Hence, the decision-making process in this regard was very heterogeneous over a study period of 20 years. As a tertiary referral center that performs large volumes of surgeries in adult patients, our current results may not apply to all institutions, especially those that have limited experience with VA-ECMO.

### Conclusions

Despite using temporary MCS with VA-ECMO, mortality rates are high in patients with normal preoperative LVEF who develop PCCS due to coronary malperfusion. However, early VA-ECMO implantation before developing profound tissue hypoxia and early coronary revascularization increase the likelihood of survival of these patients. Lactate levels are useful to define optimal timing for VA-ECMO support initiation.

## Supporting information

S1 Data (DOCX)

## Abbreviations

CI: confidence interval
CPB: cardiopulmonary bypass
IQR: interquartile range
LVAD: left ventricular assist device
LVEF: left ventricular ejection fraction
MCS: mechanical circulatory support
PCCS: postcardiotomy cardiogenic shock
ROC: receiver operating characteristic
VA-ECMO: venoarterial extracorporeal membrane oxygenation
