# Supplementary material for: Mortality in patients with normal left ventricular function requiring emergency VA-ECMO for postcardiotomy cardiogenic shock due to coronary malperfusion
Source: PLoS One. 2024 Mar 21;19(3):e0300568. doi: 10.1371/journal.pone.0300568 (PMC10956859; doi:10.1371/journal.pone.0300568)
Supplement: S1 Data — (DOCX) [file pone.0300568.s001.docx]

**Supplemental material**

Important variables for this study were defined as follows:

**1.Postcardiotomy cardiogenic shock (PCCS):** inability to wean off cardiopulmonary bypass (CPB) and/or development of intractable cardiogenic shock with inadequate mean arterial pressure and evidence of organ dysfunction secondary to insufficient perfusion pressure in the operating room (OR) or during the early postoperative period (48 hours), despite large doses of inotropic support in patients undergoing heart surgery (1,2).

**2.Mortality:** death from any cause occurring within the first 30 postoperative days in patients who were supported with VA-ECMO for PCCS (3).

**3. Bleeding:** Bleeding was defined as those patients needing re-thoracotomy to arrest the evident bleeding coming from the operative wound and/or from the surgical drains or surgical intervention in the groin due to bleeding coming from the insertion site of the VA-ECMO cannulas, hematuria, gastrointestinal bleeding and/or bleeding from the orotracheal tube, starting from the moment of VA-ECMO implantation and who needed a transfusion, in an acute clinical situation where blood loss rate of 150 ml/h with hemodynamic deterioration without any evidence of surgical bleeding (4).

**4.Acute kidney injury:** according to the KDIGO guidelines (5) it is defined as the presence of any of the following: increase in serum creatinine by ≥0.3 mg/dl (≥26.5 𝛍mol/l) within 48 hours; or increase in serum creatinine to ≥1.5 times baseline, which is known or presumed to have occurred within the prior 7 days; or urine volume <0.5 ml/kg/h for 6 hours.

**5.Acute liver failure:** development of severe acute liver injury with impaired synthetic function (INR more than 2 times the baseline) and increase in transaminases levels (10 times the baseline value) with or without encephalopathy in a patient without preexisting cirrhosis or liver disease (6).

**6.Neurologic dysfunction/insult:** neurologic impairment secondary to cerebral injury caused by stroke (ischemic or hemorrhagic), hypoxia or systemic hypoperfusion, leading to clinical manifestations of encephalopathy, such as mydriatic fixed pupils, anisocoria, seizures, absence of brain stem reflex or diabetes insipidus and confirmed employing diagnostic tests, such as cranial tomography, electroencephalogram and transcranial doppler (7).

**Supplementary references**

1. **Sylvin EA, Stern DR, Goldstein DJ.** Mechanical support for postcardiotomy cardiogenic shock: has progress been made? J Card Surg 2010;25:442–54.

2. **Doll N, Kiaii B, Borger M, Bucerius J, Krämer K, Schmitt DV, et al.** Five-Year Results of 219 Consecutive Patients Treated With Extracorporeal Membrane Oxygenation for Refractory Postoperative Cardiogenic Shock. Ann Thorac Surg 2004;77:151–7

3. **Wang L, Yang F, Wang X, Xie H, Fan E, et al.** Predicting mortality in patients undergoing VA-ECMO after coronary artery bypass grafting: the REMEMBER score. Crit Care. 2019 Jan 11;23(1):11.

4. **Cheng R, Hachamovitch R, Kittleson M, Patel J, Arabia F, et al.** Complications of extracorporeal membrane oxygenation for treatment of cardiogenic shock and cardiac arrest: a meta-analysis of 1,866 adult patients. Ann Thorac Surg. 2014 Feb;97(2):610-6.

5. **Kidney Disease: Improving Global Outcomes (KDIGO) Acute Kidney Injury Work Group.** KDIGO Clinical Practice Guideline for Acute Kidney Injury. Kidney Int Suppl. 2012;2:1–138.

6. **Lee WM, Stravitz RT, Larson AM.** Introduction to the Revised American Association for the Study of Liver Diseases Position Paper on Acute Liver Failure 2011. Hepatology. 2012 Mar;55(3):965-7.

7. **Le Guennec L, Cholet C, Huang F, Schmidt M, Bréchot N, et al.** Ischemic and hemorrhagic brain injury during venoarterial-extracorporeal membrane oxygenation. Ann Intensive Care. 2018 Dec 20;8(1):129.
